# Norepinephrine, beyond the Synapse: Coordinating Epigenetic Codes for Memory

**DOI:** 10.3390/ijms23179916

**Published:** 2022-08-31

**Authors:** Sabyasachi Maity, Raman Abbaspour, David Nahabedian, Steven A. Connor

**Affiliations:** 1 Department of Physiology, Neuroscience, and Behavioral Sciences, St. George’s University School of Medicine, True Blue FZ818, Grenada; 2 Department of Biology, York University, 4700 Keele Street, Toronto, ON M3J 1P3, Canada; 3 The Center for Biomedical Visualization, Department of Anatomical Sciences, St. George’s University School of Medicine, True Blue FZ818, Grenada

**Keywords:** norepinephrine, memory, hippocampus, beta-adrenergic receptors, synaptic plasticity, epigenome, transcription

## Abstract

The noradrenergic system is implicated in neuropathologies contributing to major disorders of the memory, including post-traumatic stress disorder and Alzheimer’s disease. Determining the impact of norepinephrine on cellular function and plasticity is thus essential for making inroads into our understanding of these brain conditions, while expanding our capacity for treating them. Norepinephrine is a neuromodulator within the mammalian central nervous system which plays important roles in cognition and associated synaptic plasticity. Specifically, norepinephrine regulates the formation of memory through the stimulation of β-ARs, increasing the dynamic range of synaptic modifiability. The mechanisms through which NE influences neural circuit function have been extended to the level of the epigenome. This review focuses on recent insights into how the noradrenergic recruitment of epigenetic modifications, including DNA methylation and post-translational modification of histones, contribute to homo- and heterosynaptic plasticity. These advances will be placed in the context of synaptic changes associated with memory formation and linked to brain disorders and neurotherapeutic applications.

## 1. Introduction

Synapses within the mammalian CNS demonstrate a remarkable capacity for activity-dependent refinement, extending from nanoscale molecular events to structural modifications. These changes serve as the cellular basis for information storage, a process known as synaptic plasticity [1,2]. Multiple forms of plasticity have been identified, ranging from short-term changes in release probability (augmentation and post-tetanic potentiation) to enduring forms of increased (long-term potentiation; LTP) and decreased (long-term depression; LTD) synaptic transmission [2,3]. Most forms of plasticity are altered by neuromodulators, including norepinephrine (NE), which acts through metabotropic receptors and associated intracellular signals to modify neuronal excitability and synaptic function. During learning and memory events, the secretion of NE activates G protein-coupled receptors (GPCRs), initiating multiple signaling cascades that broaden the parameters of a synapse’s capacity for change.

Norepinephrine acts through two separate classes of receptor (α- and β-ARs), which vary in expression levels across different cell types. Within the hippocampus, pyramidal cells and granule cells express α1, α2, β1, and β2-ARs [4,5]. In interneurons, β1-ARs are most highly expressed in CA1 and CA3 somatostatin-containing neurons and to a lesser degree in parvalbumin-positive cells. In contrast, β2-ARs demonstrate consistent expression patterns across most interneuron subtypes, with a modest elevation in cholecystokinin-expressing cells throughout the hippocampus [6]. Beta-adrenergic receptors are also found in astrocytes, where they influence a broad range of synapse functions [7]. At the level of individual synapses, β1-ARs localize to the membrane and cytoplasm but not the nucleus, whereas β2-ARs are also found in the nucleus [8]. Both α- and β-ARs express in the pre- and postsynaptic area, although this pattern demonstrates regional variation [9]). 

Generally, β-ARs are permissive for multiple forms of LTP, whereas α-1 and α-2-ARs contribute to synaptic potentiation and depression, respectively [10,11]. As these receptors demonstrate differential expression, subcellular localization, and ligand affinity, an emerging consensus is that the magnitude and direction of plasticity will be determined in part by the receptor profiles (location, density, and sub-type) and the secreted levels of NE. Given their prominent role in the LTP of glutamatergic synapses [12,13,14,15,16,17,18] and the recent evidence suggesting roles in epigenetic regulation [19], we will focus our summary on β-ARs.

## 2. Recruitment of β-ARs during Learning 

Generally, learning recruits bidirectional plasticity throughout the hippocampal circuit in vivo, and this “learning-facilitated plasticity” is subject to neuromodulation [20,21] This effect was demonstrated in adult rats, in which baseline electrical pulses applied to the Schaeffer collateral fiber pathway were paired with stimulation of the locus coeruleus. This paired paradigm initiated LTD while facilitating spatial learning, effects which required β-AR activation [20]. Similarly, spatial learning associated with the exploration of an empty hole board converted transient, rapidly decaying LTP into a long-lasting form at mossy fiber-CA3 synapses via β-AR activation [22]. These findings support the notion that exposure to novelty increases locus coeruleus activity and NE release, rendering synapses more labile and priming neural circuits for encoding.

## 3. Beta-Adrenergic Receptor Regulation of Memory

β-ARs have well-established roles in the noradrenergic effects on memory formation. These have been best demonstrated in the hippocampus, a brain region essential for encoding spatial, contextual, and semantic memories [23,24,25,26]. Consistent with this, arousing experiences included exposure to novelty, which engages the noradrenergic system, increases population spike amplitude, and bolsters LTP within the dentate gyrus [27,28]. Exposure to novelty drives neuronal activity with the brainstem locus coeruleus neurons, the primary source of noradrenergic projections throughout the brain, an effect which is partially blocked when β-ARs are inhibited. This supports the idea that the noradrenergic system promotes the transfer and processing of information in the hippocampus, acting as a physiological “gate” for information associated with novelty or arousal. 

Several types of memory have been identified with unique properties and the underlying mechanisms. Although β-ARs mediate physiological and molecular events, supporting both short- and long-term memories, most studies have implications for the long-term components of memory. Accordingly, direct injection of norepinephrine (NE) into area CA1 of the hippocampus enhanced long-term memory without affecting short-term memory [29,30]. Blocking β-ARs in area CA1 of the hippocampus prevented contextual and spatial memory formation [31,32]. Similarly, novel object recognition memory was reduced or extended through the inhibition or activation, respectively, of β-ARs in the dorsal hippocampus [33]. Direct injection of isoproterenol, a selective β-AR agonist, in the amygdala converted a normally transient contextual memory induced by weak foot shocks to an enduring form, through a process requiring calcium-permeable AMPA receptors and the serine/threonine protein kinase extracellular signal-regulated kinase (ERK), specifically during consolidation [34,35]. These findings suggest that β-ARs preferentially recruit divergent signaling pathways in support of distinct memory phases. Consistent with this, long-term associative memory was impaired by the injection of β-AR antagonists, even when administered two hours after training [36]. Based on the preferential effect on the late or enduring components of memory, noradrenaline appears to enhance the stabilization of newly formed memories [37,38], although conflicting results have been reported [39].

## 4. Facilitated Retrieval of Memories by β-ARs

Memory retrieval, the recall of established memories, is also regulated by β-ARs within the hippocampus [15,40,41]. Stimulation of the locus coeruleus promotes the retrieval of spatial memories in a food-motivated maze [15]. Additionally, direct injection of NE into the hippocampus promoted the retrieval of an inhibitory avoidance memory [41]. Further evidence for the effects on retrieval has been demonstrated in mice that genetically lack endogenous NE and adrenaline Dbh(−/−). Although the Dbh(−/−) mice were able to learn the platform location in the Morris water maze, they failed to recall the correct platform quadrant in probe trials [39]. This suggests that depletion of NE reduces spatial memory retrieval even when initial memory formation is intact. 

The recall of established memories renders them labile to facilitate content updating, after which the updated memory is stabilized; this is known as reconsolidation. β-ARs appear essential for reconsolidation as blocking their activity during the reconsolidation period induced amnesia when this memory was later tested, which is consistent with impaired reconsolidation [42]. This suggests that β-ARs may contribute in a bidirectional manner to the coordination of memory updating by regulating the reconstruction of recalled memories; however, the mechanisms remain unknown. 

Overall, these findings suggest that NE recruits a broad range of physiological effects which modulate cognition, acting primarily through β-ARs to govern information processing within the CNS. However, the persistent nature of associated synaptic changes necessitates mechanisms that are enduring in nature, beyond the transient molecular events at synapses. 

## 5. Norepinephrine and Enduring Plasticity

Foundational studies established that β-ARs engage canonical signaling pathways known to bolster the duration of LTP at glutamatergic synapses, including cAMP, cAMP-dependent protein kinase A (PKA), ERK, and mammalian target of rapamycin (mTOR) [43,44,45,46]. These myriad intracellular processes enhance neuronal signal transmission through bolstering the physiological strength of synapses. Long-term potentiation (LTP) is an activity-dependent increase in synaptic strength, representing the cellular basis for learning and memory [3,47]. LTP-like processes have been associated with memory formation in vivo [48]. Conversely, synaptic weakening or long-term depression (LTD) is a hallmark of memory loss [2,49]. The direction and magnitude of synaptic changes are determined in part by relative expression, binding kinetics, and the subcellular localization of noradrenergic receptors.

The β-AR concentration is highest in the subregions of the hippocampus [50], where it localizes primarily to pyramidal cells [4,5]. Generally, β-AR stimulation increases cell excitability, as well as synaptic strength [43,51]. LTP generated by tetanization requires β-ARs within the dentate gyrus [52,53], which was demonstrated by antagonizing the β-ARs which blocked LTP within both the medial and the lateral perforant pathways [53]. Conversely, the direct application of NE potentiates EPSPs in the medial perforant path, while depressing EPSPs in the lateral perforant path [14,54]. In vivo stimulation of the locus coeruleus induces a long-lasting (>24 h) potentiation of fEPSPs within the dentate gyrus [55]. Direct stimulation of the basolateral amygdala similarly enhances the maintenance of LTP in the DG, through a process requiring β-ARs [56]. These forms of NE-LTP require de novo translation [55,56], similar to the mechanisms required for promoting enduring memories [57].

At the mossy fiber pathway in area CA3, combining NE (or isoproterenol) with tetanization increases the induction, magnitude, and duration of LTP [13,58]. Both the transient and the enduring forms of LTP are prevented within this region by blocking the β-ARs during tetanization [59]. This mechanism is believed to be presynaptic [59], with endogenous NE increasing glutamate release, contributing to the induction of LTP. Alternatively, β-ARs are dispensable for the induction of LTP in area CA1, demonstrating a modulatory role within this subregion [12,40,60,61]. 

Beta-adrenergic signaling appears to shift the frequency-response curve as trains of low-frequency stimulation (LFS) that normally depress synaptic strength induce potentiation when the β-ARs are simultaneously activated [17,44,62]. This phenomenon recruits both PKA and ERK signaling cascades [17,44,63] and increases the “complex spikes”, which mimic the brain rhythms associated with spatial exploration [61,64]. These effects varied along the dorsal–ventral axis as exposure to early stressors amplified isoproterenol-induced LTP within the ventral hippocampus, while suppressing LTP within the dorsal hippocampus [65]. The expression of β1-ARs was upregulated in the ventral hippocampus, suggesting that juvenile stress sensitizes this circuit to NE [65]. 

Generally, the induction phase of LTP requires NMDAR activation [66], followed by a maintenance phase characterized by enhanced AMPAR retention at the synapses [67,68]. β-ARs appear to preferentially enhance the maintenance phase of LTP as their activation during tetanization generates enduring LTP, requiring mTOR and ERK signaling cascades [45,62]. These pathways converge on eukaryotic initiation factor 4E (eIF4E) and the translation repressor 4E-BP to stabilize LTP through translation regulation [45]. Collectively, these findings support the notion that NE provides a saliency signal that engages translation, enhancing the efficacy of normally sub-threshold stimuli for forming long-term memories [16,45]. 

The receptor subtype mediating these effects may depend on the tetanization protocol used. The stimulation of β2-ARs, but not β1-ARs, enhanced LTP induced using theta frequency stimulation through the phosphorylation of serine 845 (s845) on GluA1 [69]. This contrasts with previous findings demonstrating that both β1- and β2-ARs can enhance theta-LTP [44]. Resolving this will require CKO approaches to overcome the compensatory effects that may be inherent in constitutive knockout strategies.

Along with the immediate effects on synaptic modifiability, NE can initiate “metaplastic” processes that prime synapses for future plasticity [70]. The brief application of isoproterenol initiated a “silent” metaplastic switch which reduced the threshold for the induction of future LTP without detectably altering the basal synaptic transmission [71]. This silent metaplasticity expanded the timeframe for the future induction of LTP through protein synthesis-dependent mechanisms [71,72]. These results indicate that activation of β-ARs may increase the temporal window for associating distinct experiences. Similarly, β-ARs promote the heterosynaptic transfer of LTP, during which “strong” homosynaptic β-AR LTP at one synaptic pathway allows the conversion of a “weak” heterosynaptic LTP into an enduring form [46]. Collectively, these findings support a role for β-ARs in expanding the neuronal capacity for associating events that are separated in time or that differ in saliency. 

## 6. Beta-Adrenergic Receptor Recruitment of Translation

How do β-ARs regulate long-lasting glutamatergic synapse plasticity? Metaplasticity induced by NE was impaired following the inhibition of translation during the NE treatment period [73]. Puromycin incorporation confirmed an upregulation of translation following NE treatment in area CA1. Identification of the products of protein synthesis using polysomal profiling revealed that GluA1 and GluA2 AMPA receptor subunits were specifically upregulated during NE metaplasticity [73]. The selective increase in the expression of GluA1 and GluA2 suggests that the translation of the specific synaptic proteins required for potentiation is initiated downstream of β-ARs. Further investigation of the shifts in translation profiles for other proteins implicated in synaptic plasticity is required to better understand how NE impacts memory. 

Of the critical downstream effectors, the activity of cAMP-dependent protein kinase A (PKA) has been consistently associated with β-AR stimulation [71,74]. An alternative cAMP signaling pathway coupling β-ARs to translation regulation recruits the exchange protein activated by cyclic-AMP (Epac). Epac is necessary for homosynaptic β-AR LTP [75] and, unlike PKA, the inhibition of Epac impaired both homosynaptic and heterosynaptic LTP [75,76,77,78]. Accordingly, modelling suggests a functional divergence in cAMP signaling, with PKA mediating spine-restricted signaling, whereas Epac modulates the dendritically localized processes required for the expression of LTP [76]. It will be interesting to determine how the coordinated effects of PKA and Epac converge to regulate translation and if these effects are maintained in vivo during physiologically relevant processes. 

## 7. Epigenetic Regulation of Synaptic Adaptation 

**Figure 1:**
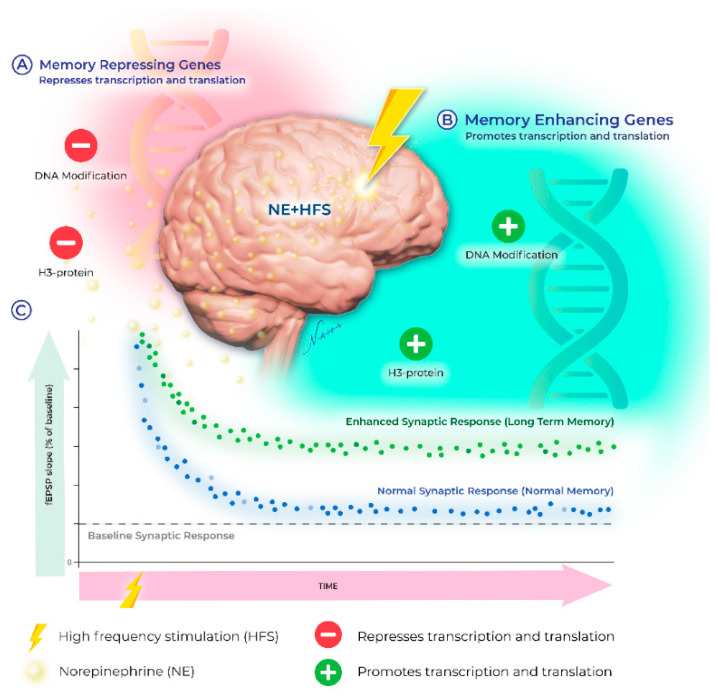
Norepinephrine engages epigenetic mechanisms permissive for plasticity. The presence of NE shifts epigenetic regulation of genes important in memory formation. NE mediated signaling upregulates the epigenetic modifications (such as, DNA methylation and H3-acetylation) of the memory enhancer genes (noted by “B” for the proteins that enhances the synaptic plasticity such as AMPA receptor subunits) but suppresses the activity of the memory repressor genes (noted by “A”). The exact mechanisms are yet to be determined. The bottom figure (C) represents the enhanced field-EPSP slope from the hippocampal CA1 region of the tri-synaptic circuit due to the paired application of NE and high-frequency stimulus (green dotted line) compared to the high-frequency stimulation only (blue dotted line). The enhanced synaptic response in presence of NE may lead to the stabilization of long-term memory. Similarly, short-term memory does not consolidate in absence of the NE.

Recently, the upregulation of transcription involving epigenetic events such as DNA methylation, histone acetylation, and histone phosphorylation have emerged as key players in the neuromodulatory landscape. Below, we provide an overview of the signaling mechanisms engaged by noradrenergic receptors and highlight the recent discoveries demonstrating that β-ARs increase synaptic modifiability through epigenetic mechanisms, which expand the cellular repertoire of the memory-related neuronal adaptations recruited by NE (see Figure 1). Transcription regulation during LTP is well established [79,80]; however, until recently, the impact of the noradrenergic system on mRNA genesis has received little attention. The differential recruitment of genomic modifications increases the computational power for the neuronal response to ongoing experiences. The expression profile of these genes is determined, in part, by epigenetic modifications which shift transcriptional output and mRNA translation dynamics. The resulting changes in expression patterns together constitute the impact of the “epigenome” on cellular function.

Early in embryological development, these epigenetic “markers” tag DNA, shifting the cellular phenotype over the individual’s lifespan through the regulation of gene expression without directly altering DNA sequences [81]. In addition to early impacts on phenotypic determination, terminally differentiated, non-dividing cells such as neurons continually acquire epigenetic modifications in response to experiences and memory-inducing events [82,83].

## 8. Overview of Epigenetic Modification

To understand how epigenetic mechanisms regulate transcription, a comprehensive structural organization of chromatin material must be considered. A long stretch of DNA wrapped around the different histone proteins constitutes the nucleosome. Nucleosomes are connected through a linker histone, H1, to form the chromatin material of the nucleus [84,85]. Inactive chromatin (“heterochromatin”) is characterized by a closed, highly compact structure which limits access to the gene transcription machinery [86]. Activity-dependent epigenetic modifications of either the DNA or the histone proteins regulate the chromatin, initiating or preventing chromatin transcription. 

Cellular DNA is methylated by the enzyme DNA methyltransferase (DNMT) through the addition of a methyl group from S-adenosyl-methionine (SAM) onto the 5′-cytosine positioned adjacent to the guanine nucleobases (CpG) [87]. The de novo DNMTs (3a and 3b) promote methylation during cell fate determination, whereas DNMT1 maintains the previous methylation markers on the DNA in the dividing cells [88]. Methylation by DNMTs represses and periodically silences gene transcription by blocking the binding of transcription factors to the regulatory sites on the DNA, thus maintaining the inactive state of the chromatin [89,90,91,92].

DNMT3A and 3B can associate with both heterochromatin and euchromatin [93,94], providing dual functions during transcription. The regulatory domain of DNA also recruits histone deacetylases (HDAC) to the DNA methylation site, where they remove an acetyl group from the histone core, further compacting the DNA, which limits transcription. DNA methylation as a dynamic mechanism initiated in response to experience-dependent events that drive increased neural activity [95,96] will be further discussed in the context of β-AR-mediated LTP below. 

The post-translational modification (PTM) of histone proteins likewise serves as a means for epigenetic regulation, independent of DNA methylation. Here, we will focus only on the role of well-characterized H3 proteins. The N-terminal tail end of histone proteins extends from the histone core, making contact with the chromatin DNA. This histone tail is the site of PTMs [97], which regulate DNA compaction and gene expression. Without PTMs, the positively charged histone proteins bind to the negatively charged DNA and thus promote the closed or inactive state of chromatin [98]. The histone tail undergoes several covalent modifications which serve as a histone “code”, including acetylation, phosphorylation, methylation, ubiquitination, and sumoylation, working synergistically to determine the chromatin structure and binding properties of the histone proteins [98,99,100].

Histone acetylation is characterized by the neutralization of the positive charges of the histone proteins by histone acetyl transferase (HAT) enzymes, which transfer one acetyl group from acetyl coenzyme A to the lysine residues of the histone tail [101,102]. Histone acetylation recruits transcription factors and RNA polymerase II, shifting transcription to its euchromatin state [103]. CREB binding protein (CBP) is a well-characterized example of a HAT associated with the regulation of transcription during learning and memory [104,105,106,107,108]. Finally, histone methyl transferases (HMTs) transfer methyl groups from S-adenosine methionine to the lysine residue of the histone tail [109]. 

Histone methylation is a reversible process, and it has a role in the assembling of heterochromatin and the maintenance of balance between the hetero- and the euchromatin [110,111]. HMTs are capable of bidirectional regulation of transcription competency with H3-lys 4 methylation-promoting and H3-lys 9 methylation-suppressing transcription, respectively [112]. H3 Phosphorylation on Ser 10 is mediated by ribosomal protein S6 kinase 2 (RSK2), a downstream signaling molecule to the aurora kinase family member [113,114].

## 9. Enzymatic Regulation of the Epigenome

With the recent discovery of broad-spectrum small molecule inhibitors, pharmacological manipulations of DNA methylation and histone acetylation can achieve activation of the repression of genes, leading to the bidirectional control of various epigenetic modifications in research as well as in the treatment of diseases. For example, most commonly available DNMT inhibitors (5-AZA and zebularine) are incorporated during the DNA replication process, leading to interference with the covalent binding of DNMTs with DNA and the demethylation of gene [115,116,117,118,119]. Similarly, specific cell-permeable inhibitors of HATs (such as p300/CBP and PCAF) and HDACs (such as class I and II HDAC) have also been identified to regulate gene expression through interfering with the post-translational modification of histones. For example, C646 is a reversible HAT inhibitor for p300/CBP, and trichostatin A (TSA) inhibits both class I and class II of the HDACs [120,121,122,123,124]. Histone phosphorylation is inhibited via the specific Aurora kinase B inhibitor AZD1152 [125]. Clinical trials using these epigenetic inhibitors show promising results in the treatment of diseases, including myelodysplastic syndrome (MDS) and other leukemias [126].

## 10. Signaling Pathways Regulating the Epigenome

Various cytoplasmic signaling molecules have been shown to influence the downstream epigenetic mechanism in neuronal tissue. The ERK/MAPK pathway, which integrates multiple post-translational modifications of histones, has been heavily implicated in learning and memory processes [127,128,129,130]. For example, ERK phosphorylates the transcription factor CREB [131,132], leading to the recruitment of transcription co-activator CBP through associated HAT activity [108]. In addition to H3 acetylation, ERK/MAPK signaling also drives histone (H3) phosphorylation through mitogen- and stress-activated protein kinase 1 (MSK1) [133,134,135]. Interestingly, HDAC inhibitors enhance object recognition memory through the upregulation of transcription, an effect that is blocked through the application of PKA inhibitors [136]. 

DNA methylation is also subject to regulation by the ERK/MAPK pathway, as methylation was reduced following the intrahippocampal injection of an NMDA receptor antagonist [137,138]. These findings support a model in which cell surface receptors (e.g., NMDARs) engage downstream ERK/MAPK signaling to initiate epigenetic modifications. Neurons similarly recruit epigenetic mechanisms to regulate the genes required for various forms of synaptic plasticity. For example, neurons express transcription co-repressors, such as REST binding protein, SIN3A, and REST co-repressor (Co-REST) [139,140,141]. Moreover, histone acetylation or deacetylation and DNA methylation are required for REST-dependent gene silencing; REST/SIN3A repressor complexes interact with HDAC1, whereas the REST/Co-REST complexes are associated with HDAC2 [142,143], providing the basis for the epigenetic regulatory mechanisms in the CNS. 

HDACs are of two types primarily, class I and class II with distinct specificity on intracellular location leading to the recruitment of diverse signaling pathways to control cellular function. Class II HDACs again could be of class IIa (HDAC4, 5,7, 9) or class IIb (HDAC6, 10). While the class I HDACs (HDAC 1, 2, 3, and 8), are located in the nucleus predominantly, the class II HDACs (HDAC 4, 5, 6, 7, 9, and 10;) are expressed in both the nucleus and cytoplasm, shuttling between both the compartments [144].

Although both of the classes of HDACs are involved in neurodevelopment, research on assessing synaptic plasticity and memory formation in mammalian brain has extensively studied the use of class I HDACs (particularly HDAC2), but not class II HDACs. The reason could be the inability of the class II HDACs to inhibit the cellular process leading to the learning and memory due to the week enzymatic action by themselves. However, to overcome this weakness, class II forms a enzymatic complex with Class I and thus perform the deacetylase activity [145]. Having said that, stimulation of either class of HDACs too have opposing effects on adult learning behavior and memory formation reported by a few studies [146,147,148,149,150].

Interestingly, hippocampal knock out of HDAC3 (class I) elevates HDAC4 (class II) expression, complicating the misinterpretation of the effect of HDAC3 inhibition on memory formation [151]. A study reported that a postnatal forebrain KO of HDAC4, but not HDAC5 (although both are class II HDACs) impairs learning and memory in mice [146]. Moreover, Sando and others [148] reported that a truncated form of HDAC4 represses the genes of synaptic plasticity leading to deficiency of learning and memory. Consistent with the above behavioral studies, electrophysiological evidences also indicate that postnatal neuronal KO of HDAC4 (but not the HDAC5) in the forebrain reduces hippocampal LTP at the CA1 region [146]. These studies demonstrates an intricate and yet-to-be explored relationship between the impact of class II HDACs (4 or 5 mainly) KO on synaptic plasticity and learning and memory performance in mammals.

## 11. Epigenetic Regulation Associated with Memory

Early studies reported that hippocampus-dependent learning alters DNA methylation [138,152]. More specifically, contextual fear memory was prevented by DNMT inhibition, and forming a new fear memory was positively correlated with the upregulation of DNMT gene expression in the hippocampus [137,138,153]. Moreover, the inhibition of methylation alters the epigenetic marker patterns for specific memory-related genes linked to synaptic plasticity, including reelin, BDNF, and protein phosphatase 1 (PP1) [138,154]. Frankland and others [155] found that inactivation of the anterior cingulate cortex (ACC) at 18 and 36 days (a time point consistent with remote memory), but not 1 or 3 days, post-training (recent memory) reduces fear memory retrieval. This observation indicates that consolidation of this fear memory between 3 and 18 days induces cortical DNA methylation, perhaps to support transfer of a memory trace between the hippocampus and the cortex. DNA methylation was required for the maintenance and stability of these remote memories as application of DNMT antagonists 30 days after initial memory formation reduced the displays of remote fear memories [156]. This suggests that epigenetic mechanisms impact memory storage for extended periods following memory acquisition. 

Similarly, histone acetylation regulates the recruitment of transcription factors which dictate the expression patterns of memory enhancer or repressor genes, which in turn govern synaptic changes during memory formation [138,157,158,159]. Similar to DNMTs, H3-methylation of the Zif268 gene is increased following contextual fear conditioning [160]. This suggests the interesting possibility that selective methylation of DNA in tandem with histone modifications co-regulate genetic output to support memory processes. Consistent with this, shifts in epigenetic markers have been linked to neurodegenerative diseases, characterized by impaired cognitive function including working and long-term memory deficits [133,157,161,162,163]. 

In the simplified CNS of aplysia, it was shown that 5-HT induces acetylation of H3 and H4 proteins at the C/EBP promoter region during long-term facilitation (LTF) [164]. Inhibiting HDACs with TSA reduced the number of serotonin pulses required to generate LTF, which typically requires multiple pulses to achieve. HDAC inhibition also enhances the formation of LTP, using a “weak” sub-threshold stimulus [19]. Specifically, when a sub-threshold stimulus was paired with the application of an HDAC inhibitor, a long-lasting LTP was induced which mirrored the molecular requirements for multiple train LTP, including PKA/CREB transcription [108]. 

Further evidence for the importance of CBP HAT activity was demonstrated in mice haploinsufficient for CBP+/−. Mice lacking a single copy of CBP showed impaired long-lasting or “late” (L-LTP) with intact “early” LTP (E-LTP) [105]. Impaired L-LTP in CBP+/− mice was restored by HDAC inhibition, indicating that impaired LTP in CBP+/− mice is due to deficient HAT activity. Consistent with a preferential role in long-lasting LTP, the application of an HDAC inhibitor enhanced the forskolin-induced expression of genes linked to memory formation (such as Nr4a1) [165], suggesting a central role in the mechanisms supporting L-LTP. These studies indicate that the acetylation and deacetylation of histones plays a major role in synaptic plasticity in multiple brain regions critical for memory. 

A requirement for epigenetic regulation extends beyond the fear memory-based paradigm. Both classical conditioning (using an eye-blink conditioning protocol) and novel object recognition are associated with the upregulation of H3 acetylation. Memory performance is bolstered in these tasks following HDAC inhibition [166]. The manipulation of HDAC activity appears to facilitate the conversion of short-term memories to enduring forms as pairing a weak training stimulus (short-term memory inducing) with HDAC inhibitor treatment generated a long-term memory [167]. This is in line with the physiological observation that a single train of high-frequency stimuli (analogous to a weak memory) in the hippocampal CA1 region was converted to a transcription-dependent, enduring form of LTP when applied during HDAC inhibition [19]. Therefore, histone acetylation influences the onset and consolidation of synaptic changes subserving long-term memory. This further suggests that HDAC acts as a negative constraint on memory formation [168], which was confirmed through the overexpression of the HDAC2 gene, which impaired memory, whereas the reduction in HDAC2 enhanced LTP [169].

Another PTM, histone phosphorylation, appears to regulate chromatin dynamics and memory genesis [159]. The mitogen- and stress-activated protein kinase 1 (MSK1) plays a crucial role in histone phosphorylation. MSK1 knockout mice show impairment of long-term spatial and contextual fear memory formation in the hippocampus and a deficiency in histone phosphorylation in the hippocampus after fear training [135]. HDAC inhibitors, however, failed to rescue the memory deficit in the MSK1 knockout mice. This contrasting finding implies potential crosstalk between histone acetylation and phosphorylation through a common regulator. In addition to MSK, IκB kinase (IKK) complex also regulates histone phosphorylation in the hippocampus [170]. These studies highlight the role of histone kinases in memory formation but require further investigation to determine how these regulatory processes interact to optimize memory regulation. 

## 12. Norepinephrine Engagement of Epigenetic Regulation 

Non-neuronal tissues recruit epigenetic modification when NE is applied [171,172], and stimulation of adrenergic receptors recruits the histone acetylation and deacetylation epigenetic mechanism in non-neuronal tissues [173,174]. Within the brain, dynamic epigenetic remodeling in response to dopamine, acetylcholine, and glutamate has been observed [175]. A canonical example is the phosphorylation of CREB, which initiates nuclear signaling pathways capable of generating mRNA transcripts supporting hippocampal plasticity [176]. Phosphorylated CREB recruits CBP-HAT for transcription of genes by removing transcriptional repressors and thus enhances synaptic plasticity and memory [177]. 

Endogenous NE, similar to the selective stimulation of β-ARs, induces a long-lasting, protein synthesis-dependent form of LTP, which requires ERK signaling [19]. ERK also has an established capacity for nuclear translocation and gene regulation. Accordingly, Maity and others [19] tested whether transcriptional mechanisms are engaged during NE-LTP. The inhibition of transcription (by Act-D or DRB) decreased the magnitude and duration of L-LTP induced through pairing weak LTP with NE [19], suggesting that a transcription-dependent mechanism is activated downstream of β-AR in the hippocampus. Further characterization revealed that NE regulates transcription by activating DNA methyl transferase, recruiting histone acetyltransferase CBP/p300 and inhibiting histone deacetylases [19]. Likewise, histone phosphorylation was also required as inhibition of Aurora kinase-B impaired NE-LTP, whereas levels of phosphorylated histone H3 were increased following the induction of NE-LTP. These effects were similarly observed for heterosynaptic plasticity as transcriptional regulation in the forms of both histone acetylation and DNA methylation were required [178]. These epigenetic modifications may regulate the expression of mRNAs coding for plasticity-related proteins, including AMPA receptor subunits, providing a link between the epigenetic and transcription regulation associated with long-term memory and L-LTP [79,179,180,181]. 

How do β-ARs convert cell-surface signals to epigenetic changes? One possibility is through the convergent regulation of ERK (extracellular signal-regulated protein kinase) and mTOR (mammalian target of rapamycin) pathways, which may simultaneously engage the transcription of memory-enhancing genes, while regulating protein synthesis at the synapse [78,182]. The transcellular coordination of these processes provides a means for generating the local proteins required for the immediate support of synaptic strength gains, while providing the permissive signals at the level of the nucleus to facilitate the maintenance of the potentiation over time. Evidence supporting this coordinated effect between local synaptic potentiation and ongoing epigenetic changes was demonstrated through the inhibition of HDAC, which increased the CBP-HAT, leading to increased expression of CRE reporter genes by cAMP [165,183]. Similarly, H3 acetylation and transcription were also significantly enhanced during β-AR-mediated synaptic plasticity [19,77]. 

**Figure 2:**
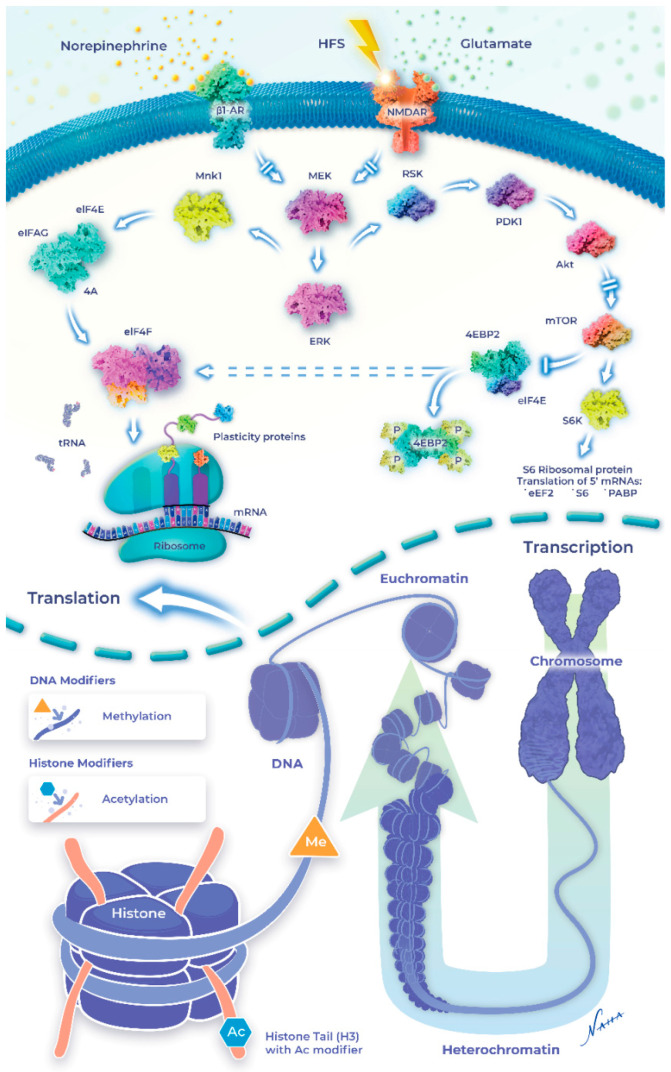
Signaling pathway linking beta-adrenergic receptors to regulation of the epigenome. Norepinephrine couples to ERK signaling which regulates cytoplasmic protein synthesis and initiates downstream signals that alter epigenetic markers. During NE-LTP, NMDARs are similarly recruited and act synergistically to upregulate translation and alter the epigenome. Intracellular signaling mechanisms also recruit (through unknown pathway) the epigenetic mechanisms such as DNA methylation and histone acetylation to increase the transcription of memory enhancing genes leading to increased synthesis of plasticity related proteins. The nucleosome is unwrapped in the presence of the NE-induced signaling leading to expose the site of the epigenetic modification at the DNA and the Histone tails. Therefore, the condensed heterochromatin becomes activated euchromatin and thus facilitates further process of transcription. DNMT (DNA n-methyl transferase) and HAT (Histone acetyl transferase) are the enzymes responsible for the tagging of the DNA and histone proteins(H3) by adding one methyl molecule and one acetyl molecule to the DNA and H3 proteins respectively. Other post-translational modifications such as histone phosphorylation, ubiquitination, methylation, etc., are not shown in this figure to avoid the clumsiness.

Interestingly, NE can alter the chromatin structure to make it either accessible or inaccessible for DNA methylation in addition to post-translational modifications (i.e., acetylation and phosphorylation) of the core histone proteins (Figure 2). β-AR-LTP is reduced when DNA methylation is inhibited by AZA or ZAB, which would de-repress the memory suppressor genes [19]. Immunohistochemical analysis confirmed that a β-AR agonist increased H3K14 acetylation in the mouse hippocampus [133]. β-AR stimulation also stabilizes heterosynaptic LTP through DNA methylation and histone acetylation [77]. As mentioned, local protein synthesis appears to be sufficient for sustaining β-AR-LTP; however, questions remained as to whether a late (>3 h) component of β-AR LTP requires the upregulation of mRNA synthesis through epigenetic mechanisms. Evidence supporting this idea was provided through HDAC inhibition, which transiently increased the quantity of acetylated H3, converting a transient form of LTP into an enduring form [108]. Interestingly, recent findings failed to detect the heterosynaptic enhancement of LTP when β-AR stimulation was paired with TSA [77]. Taken together, these studies suggest that HDAC inhibition may recruit the same signaling pathways as histone acetylation, although the pro-LTP effects may be limited to only those synapses active during HDAC inhibition.

Interestingly, neuromodulator dopamine along with glutamate converges on the regulation of H3pS10 in the mouse dentate gyrus [175]. H3 acetylation at Lys14 is coupled to H3 phosphorylation at serine-10 (H3pS10), activating transcriptional factors [184]. Similarly, phosphorylation of H3 at serine-10 is increased when NE is paired with tetanization, generating persistent LTP that is abolished in the presence of AZD11, an H3 phosphorylation inhibitor [19,77]. These data suggest that increased H3 serine-10 phosphorylation is recruited downstream of cell surface receptors, including β-ARs, to alter the duration and amplitude of synaptic potentiation. Recent evidence suggests that the effects of β-AR-mediated histone modification appear restricted temporally to the consolidation phase of fear memory [185]. Collectively, these results identified transcriptional regulation at the level of the epigenome as a key mechanism supporting the long-term modification of the synapses required for memory processes downstream of β-ARs. A simplified diagram is shown to indicate NE-induced intracellular signaling and the epigenetic modifications in synaptic plasticity and learning and memory (Figure 2).

## 13. Clinical Implications for Epigenetic Modification

Epigenetic modification during β-AR-mediated long-term synaptic plasticity has important implications for disorders characterized by excessive (post-traumatic stress disorder; PTSD) or impaired (Alzheimer’s disease; AD) memory function. The NE concentration in human cerebrospinal fluid is significantly increased in PTSD patients [186], and the β-blocker propranolol has shown limited success in reducing the probability of developing PTSD [187]. This raises the interesting question of whether the selective targeting of epigenetic mechanisms downstream of β-ARs is required for reducing the symptoms and involuntary recall associated with PTSD. Given the perseverative nature of PTSD, it is likely that the epigenetic mechanisms required for long-term memory are uniquely impacted in this disorder and correspond to those recruited by β-AR stimulation. 

Impaired noradrenergic neurotransmission has likewise been implicated in many cognitive disorders, particularly neurodegenerative diseases such as Alzheimer’s disease [188]. Age-related impairments of the LC system and noradrenergic function are implicated in memory loss [189]. Along with these, normal epigenetic modifications are disrupted in memory-related cognitive disorders [190], and AD, which is characterized by neuron and synapse loss, shows dysregulation of histone acetylation [191,192,193]. Accordingly, HDAC inhibitors should provide novel therapeutic options for restoring cognitive function in age-related brain disorders.

As multiple signaling molecules converge on nuclear epigenetic mechanisms, important questions remain surrounding the optimal methods for harnessing these mechanisms for therapeutic purposes. Given the epigenetic regulation of gene expression during β-AR-LTP, the changes in acetylation patterns of H3 downstream of β-ARs could represent a therapeutic opportunity. A major limitation surrounds the effects of NE and β-AR agonists on non-neuronal tissues throughout the body. Most notably, NE has a powerful effect on heart rate and blood pressure, which could limit the use of these approaches in populations with pre-existing heart conditions. It will be important to test whether the manipulation of the epigenetic regulatory processes linked to β-ARs can be targeted without inducing the potentially negative effects of β-AR stimulation on heart function.

## Abbreviations

α-AR	Alpha-adrenergic receptor
β-AR	Beta-adrenergic receptor
CNS	Central nervous system
EPSP	Extracellular postsynaptic potential
ERK	Extracellular signal-regulated kinase
G-protein	Guanine nucleotide-binding regulatory protein
HFS	High-frequency stimulation
LC	Locus coeruleus
LFS	Low-frequency stimulation
LTD	Long-term depression
LTM	Long-term memory
LTP	Long-term potentiation
mTOR	Mammalian target of rapamycin
NE	Norepinephrine
NMDA	N-methyl-D-aspartate
NST	Nucleus of the solitary tract
PKA	cAMP-dependent protein kinase
PTSD	Post-traumatic stress disorder
STM	Short-term memory
